# The Prognostic Role of MET Protein Expression Among Surgically Resected Non-small Cell Lung Cancer Patients: A Meta-Analysis

**DOI:** 10.3389/fonc.2019.01441

**Published:** 2019-12-20

**Authors:** Guangzhi Ma, Yunfu Deng, Wenjie Chen, Zhenkun Liu, Cheng Ai, Xuebing Li, Qinghua Zhou

**Affiliations:** ^1^Lung Cancer Center, West China Hospital, Sichuan University, Chengdu, China; ^2^Department of Thoracic Surgery, West China Hospital, Sichuan University, Chengdu, China; ^3^Tianjin Key Laboratory of Lung Cancer Metastasis and Tumor Microenvironment, Tianjin Lung Cancer Institute, Tianjin Medical University General Hospital, Tianjin, China

**Keywords:** MET protein, prognostic role, resected, non-small cell cancer, meta-analysis

## Abstract

**Objectives:** MET protein expression has been reported to be in relevance with the survival of NSCLC patients in various studies, yet the results were inconsistent. The purpose of our study set out to determine the prognostic role of both c-MET and p-MET expression among NSCLC that underwent surgical resection.

**Methods:** Data were obtained from retrospective cohort studies by searching on PubMed, Cochrane Library, EMBASE and Web of Science, and a meta-analysis was performed to assess the prognostic role of MET expression among NSCLC.

**Results:** Totally 18 literatures including 5,572 surgically resected NSCLC cases staged I-IV were included for data synthesis. The positive rate of c-MET and p-MET was 1,753/4,315 and 135/1,257. The pooled hazard ratios (HRs) regarding c-MET and p-MET expression for overall survival (OS) was 1.623 (95% CI: 1.176–2.240, *p* = 0.003) and 1.710 (95% CI: 0.823–3.533, *p* = 0.15), respectively. Subgroup analysis results on Asian (HR = 2.115, *p* < 0.001), adenocarcinoma (HR = 2.220, *p* < 0.001) and rabbit polyclonal antibodies (HR = 2.107, *p* < 0.001) etc. were also indicative.

**Conclusion:** C-MET over-expression among NSCLC patients that underwent surgical resection is a prognostic factor that indicated adverse survival on OS. Whereas, p-met didn't appear to have an impact on the prognosis of NSCLC. The studies are need and the topic could be re-valued by then.

## Introduction

Lung cancer remains the leading cause of cancer incidence and mortality worldwide, accounted for ~1.8 million deaths in 2018 (1). Among which statistically 85–90% of lung cancer cases were non-small cell lung cancer, or NSCLC based on pathologic classification (2). With the development of target-therapy and immunotherapy, alternatives to deal with NSCLC posterior to *en bloc* resection is comprehensive (3). Yet besides the efforts to improve therapeutic methods and diagnostic accuracy, the outcomes of NSCLC patients remains unsatisfactory (4, 5).

MET protein, also known as hepatocyte growth factor receptor (HGFR), has been characterized as a high affinity transmembrane receptor tyrosine kinase (RTK) which is encoded by its homologous oncogene *MET* (6, 7). Being firstly recognized in osteosarcoma derived cell-lines, MET was subsequently identified to have over-expressed in various malignances including NSCLC (8, 9). When c-MET binds to its homologous ligand HGF, the intracellular tyrosine residues of the RTK became activated via auto-phosphorylation (p-MET) (10). P-MET accordingly triggers its downstream pathways such as PI3k-Akt, Ras-MAPK, and STAT3, which physiologically promotes tissue growth, vascularization, and healing (11, 12). Whereas, the aberrant expression of MET would result in tumorigenesis and development of various malignancies, including NSCLC (13, 14). The mechanisms that led abnormal HGF/c-MET signaling were either *c-MET* amplification, mutation or MET/HGF overexpression, and among which MET over-expression most frequently occurred (15, 16). Prior studies have noted alterations regarding HGF/c-MET signaling played a key role among NSCLC patient that acquired resistance to first generation EGFR-TKIs due to its underlying interactions with EGFR pathways (17, 18). In addition, targeting *MET* as well as MET upregulation via either TKIs or MET-antibodies has already become a novel strategy to challenge NSCLC patients with metastatic disease (19–22). Hence, understanding the impact of c-MET/p-MET expression on NSCLC survival should be highlighted. As primary c-MET/p-MET expression status of NSCLC patients was majorly from resected-specimen tumors via immunohistochemistry (IHC), patients that received surgical therapy was our main concern.

To date literatures has emerged with inconsistent conclusions on the prognostic role of MET among NSCLC. C-MET expression was thought to be a favorable biomarker in various studies (23–25), yet others suggested the opposite (26–28). In addition to some studies, neither c-MET nor p-MET expression was related with NSCLC survival (29, 30). Thus, due to the contradictory results from previous studies, we herein set out to conduct a systematic review as well as meta-analysis by summarizing current existing data to examine the survival implications of MET over-expression among lung cancer patients that underwent surgical resection.

## Materials and Methods

### Literature Search

Two reviewers (GM and YD), respectively, conducted electronic search on PubMed, Cochrane Library, EMBASE, and Web of Science for relevant studies up till July 15th, 2019, with the beginning date unlimited. The search terms were as followed: “MET” or “Mesenchymal Epithelial Transition factor” or “Hepatocyte growth factor receptor” and “Non-small cell lung cancer” or “NSCLC” or “Pulmonary carcinoma” or “lung cancer” and “Prognosis” or “Outcomes” or “Survival.”

### Inclusion Criteria

Eligible studies was required to be in compliance with the following criteria: (1) NSCLC studies, all included participants should be NSCLC patients that underwent surgical resection; (2) MET expression was examined of each resected specimen, with the correlation between MET expression and NSCLC survival been reported; (3) Hazard Ratio (HR) was clearly displayed and feasible for HR synthesis, according to methods described by Parmar et al. (31), Williamson et al. (32), and Tierney et al. (33); (4) Study designs include: randomized controlled trial (RCT) and cohort study.

### Exclusion Criteria

Articles were omitted from further consideration if: (1). Systematic review or review; (2) Preclinical studies, such as laboratorial or *in vitro* studies; (3) Case reports; (4). Studies of which survival data (including survival curves yet without HRs reported) unavailable for further calculations.

### Data Extraction

Basic information of each eligible study was extracted as followed: name of first author, publication year, country, demographic characteristics (number of patients, gender, and median age), smoking status, pathology, and tumor stage, antibody applied for MET immunohistochemical (IHC) staining, cut-off value of MET over-expression and reported HRs (representing prognosis) for meta-analysis.

The primary data eligible for calculation and results-pooling was hazard ratios (HRs) reported from either multivariate or univariate Cox hazard regression analysis for overall survival (OS). Literatures of eligibility was filtered by two authors (GM and YD) individually, with any discordance being revised and re-assessed.

### Quality Evaluation

The Newcastle–Ottawa Scale (NOS) criterion was adopted for quality assessment of included studies (34). The criteria covered three aspects of each study: (1) selection of subject: 0–4; (2) subject comparability: 0–2; and (3) survival: 0–3. The scope regarding the final score ranged between 0 and 9, literature with six or more were reckoned feasible for data incorporation and any scored no <7 were considered of good quality. Two reviewers independently carried out quality evaluation of each study.

### Statistical Analysis

Data calculation and meta-analyses were performed via STATA (version 12.0, STATA Corporation, Texas, USA). LogHRs reported in the literature were prior used for HR pooling, otherwise HRs with 95% confidential intervals (CIs) were considered for data syntheses. Multivariate analyses data were prior adopted if multivariate and univariate survival analyses were both conducted. Adjusted HRs was used when unadjusted/adjusted HRs both existed. Chi-square based *Q*-test and *I*^2^ statistic test were performed to value heterogeneity regarding the pooled HRs (35). The Mantel-Haenszel method or fixed-effect models (36) were adopted when study heterogeneity wasn't statistically considered significant (*I*^2^ < 50% or *P* > 0.10) whereas random-effect models were applied for calculation in order to minimize potential influence of heterogeneity on pooled results. Apart from random-effect model, sensitivity analysis by leave one out procedures was also processed uncovering the potential source regarding heterogeneity of pooled results (37). Publication bias were conducted in accordance with Begg's methods (38). Publication bias was reckoned significant when *P*-value was <0.05.

## Results

### Study Selection

Our initial literature search retrieved 1,151 studies (after duplicates removal) in total. Abstracts of each identified publication was discreetly read and screened. Studies were removed due to the reasons as followed: Reviews or systematic review (*n* = 124), case reports (*n* = 158), irrelevant topic or fundamental observations (*n* = 807). Totally 62 potential studies of eligibly were obtained and scrutinized. Then 45 of which were omitted owing to the following reasons: 34 studies focused on irrelevant topics such as MET gene expression and alterations, 11 remaining studies whose data were either survival curve or illegible of HR estimation. Two studies conducted by Sun et.al included overlapped patients (28, 39). To limit potential risk of bias, we omitted the publication with lesser participants. Hence, altogether 17 studies eventually met our criteria of inclusion and were capable of data extraction as well as meta-analyses.

**Figure 1:**
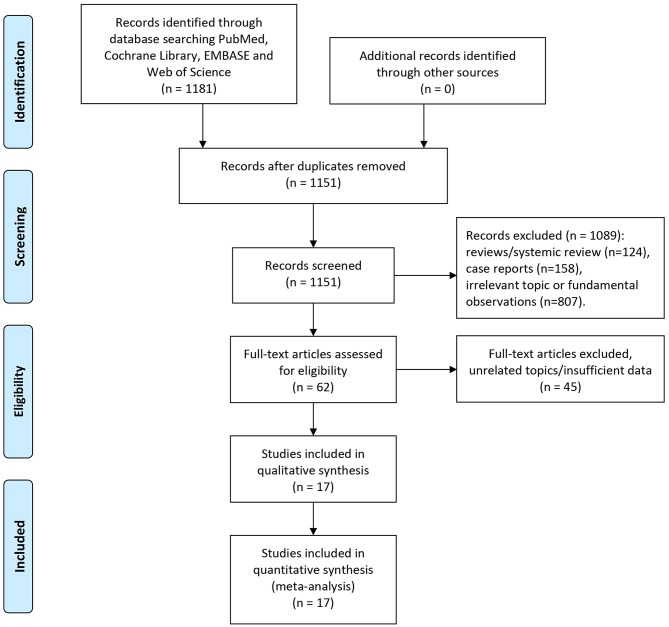
Flow diagram of study selection.

Summarized process of literature selection was displayed in the flow chart of Figure 1.

### Baseline Characteristics

In all, our topic was demonstrated in 17 studies. Among which Asian studies dominated in quantity, including six from Japan (26, 29, 30, 40–42), five from China (23, 39, 43–45), and two from Korea (27, 46). Caucasian patients that were either from Sweden (25), Netherlands (47), Poland (48), or Australia (24) comprised the rest population of included studies. Totally 4,315 NSCLC patients staged between I-IV that underwent surgical resection were assessed of c-MET expression, while 1,257 participants had p-MET evaluation. Immunohistochemical staining (IHC) was performed on each corresponding NSCLC tissue to value c-MET/p-MET expression, and the rabbit derived antibodies accounted for the majority of antibodies to against MET protein. All studies mentioned HRs that were feasible for data-pooling. MET over-expression were determined in accordance with certain measurements that had varied threshold values such as H-score or H intensity. All studies scored no <6 with reference to the NOS quality criterion, hence applicable for our meta-analysis.

Baseline information was listed on Table 1.

**Table 1:** Baseline characteristics of the included publications.

**References**	**Country**	**Median age**	**N(F/M)**	**Smoking (S/NS)**	**Histology**	**Stage**	**MET type**	**Antibody**	**Cut-off value**	**MET high**	**MET low**	**HR estimation**
Tsakonas et al. (25)	Sweden	66.5	653 (316/337)	589/64	NSCLC	IA–IIIB	c-MET	PharmDx	H-score ≥ 20	336	117	Multi
Zhang et al. (45)	China	60.4	86 (44/42)	29/57	ADCC	I–IV	c-MET	RM (SP44)	Staining score ≥ 2+ (50%)	54	32	Multi
Kim et al. (46)	Korea	NR	311 (140/171)	109/202	ADCC	IB–IIIA	c-MET	RM (SP44)	Staining score ≥ 2+ (50%)	141	170	Multi
Tran et al. (24)	Australia	67 (–)/ 69 (+)	271 (98/173)	211/9	NSCLC	I–III	c-MET	RM (SP44)	Staining score ≥ 2+ (50%)	248	23	Multi
Tong et al. (44)	China (HK)	66	687 (223/464)	395/223	NSCLC	I–IV	c-MET	RM (SP44)	Staining score ≥ 2+ (50%)	230	457	Uni
Wang et al. (43)	China	57	117 (33/84)	43/74	NSCLC	I–IV	c-MET	R*	H-score ≥ 1.9	36	81	Multi
Huang et al. (23)	China	62	102 (29/73)	47/55	NSCLC	I–IV	c-MET	RM	H-score ≥ 60	52	50	Multi
Sun et al. (39)	China	56.2	183 (42/141)	117/66	ADCC/SCC	I–IV	c-MET	R*	Staining score > 3	123	60	Multi
Tsuta et al. (30)	Japan	61.7	906 (332/574)	416/490	NSCLC	I–IV	c-MET	RM	stained cells ≥ 10%/MA	196	687	Uni
							p-MET	RM	stained cells ≥ 10%/MA	51	829	Uni
Tachibana et al. (29)	Japan	64	106 (55/51)	55/51	ADCC	I–III	c-MET	RP	H intensity ≥ 2+	30	76	Uni
Park et al. (27)	Korea	62	380 (72/308)	279/101	ADCC/SCC	I–IV	c-MET	RP	H-score ≥ 4	52	328	Multi
Dziadziuszko et al. (48)	Poland	63	174 (39/135)	165/9	NSCLC	I–IV	c-MET	RM (SP44)	H-score > 60	83	91	Uni
Onitsuka et al. (26)	Japan	68.5	183 (81/102)	NR	ADCC	I–III	p-MET	M*	IHC Allred score ≥ 3	12	171	Multi
Ruiz et al. (47)	Netherlands	NR	168	NR	NSCLC	I–III	p-MET	NR	H-score > 5	72	96	Multi
Masuya et al. (42)	Japan	NR	88	NR	NSCLC	I–III	c-MET	RP	H intensity > grade 1	36	52	Uni
Tokunou et al. (41)	Japan	59	131 (58/73)	NR	ADCC	I–IV	c-MET	RP	Stained bundles ≥ 1/MA	69	62	Multi
Takanami et al. (40)	Japan	61	120 (51/69)	NR	ADCC	I–IV	c-MET	RP (C-28)	Stained cells ≥ 25%/MA	67	53	Multi

N, Number of patients; F, Female; M, Male; S, Smoker; NS, Non-smoker; NSCLC, Non-small cell lung cancer; ADCC, Adenocarcinoma; SCC, Squamous cell carcinoma; RM, Rabbit monoclonal; RP, Rabbit polyclonal; R*, Rabbit; M*, Monoclonal; NR, Not reported; MM, Mouse monoclonal; MA, Microscopic area; Multi, Multivariate analysis; Uni, Univariate analysis.

### Results From Meta-Analyses

**Figure 2:**
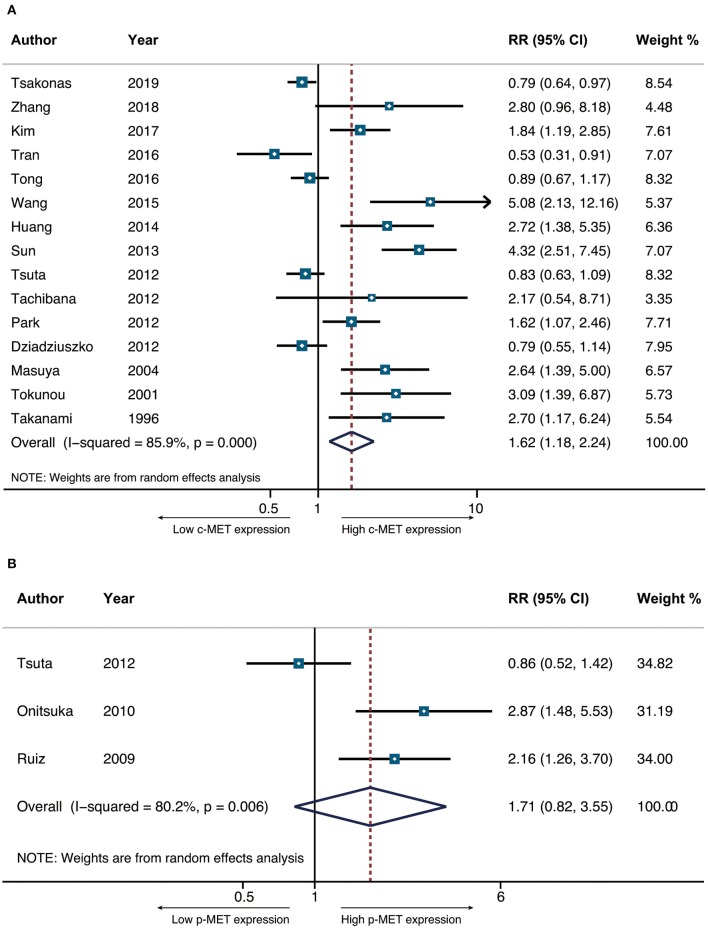
The pooled hazard ratio (HR) for OS in surgically resected NSCLC patients that had positive c-MET expression **(A)** and p-MET expression **(B)**.

The primary end-point of surveillance among included studies was OS. The correlation between MET and outcomes was determined in accordance with combined HRs and related intervals. As a result, the prognostic role of total MET protein or c-MET expression was analyzed in 15 studies of which the combined HR was 1.623 (95% CI: 1.176–2.240, *p* = 0.003), indicating an adverse impact of c-MET expression on NSCLC prognosis. Heterogeneity was significant (*I*^2^ = 85.9) thus random-effect model was adopted (Figure 2A). With regard to activated c-MET or p-MET, however, apart from potential heterogeneity (*I*^2^ = 80.2, *p* = 0.003) when combining the three related studies, the pooled result for OS (HR = 1.710, 95% CI: 0.823–3.533, *p* = 0.15) was neither indicative (Figure 2B).

### Subgroup Analyses

Subgroups were performed in terms of demographic distributions and characteristics from all eligible studies. Subgroups were stratified by (1) Regions (Asian/Non-Asian/Japanese/Chinese); (2) Histology (Adenocarcinoma); (3) Antibodies for IHC (Rabbit monoclonal/Rabbit polyclonal); (4) MET evaluation (H-score); and (5) Derived data (via multivariate analysis or univariate analysis).

#### Asian/Non-Asian/Japanese/Chinese

Totally 12 studies were conducted in Asia, and the pooled HR for OS was 2.115 (95% CI 1.440–3.108, *P* < 0.001, *I*^2^ = 83.5%). The pooled HRs via random-effect models from five Japanese studies and five Chinese studies was 1.985 (95% CI 0.970–4.058, *P* = 0.06) and 2.627 (95% CI 1.123–6.146, *p* = 0.026, *I*^2^ = 90.1%), respectively. With respect to non-Asian patients, the combined HR for OS from four studies was 0.901 (95% CI 0.586–1.387, *p* = 0.637), with random-effect model due to its significant heterogeneity (*p* = 0.002, *I*^2^ = 80.0%).

#### Adenocarcinoma

The prospect of our present study was to examine the prognostic role of MET expression on multiple NSCLC types. Yet only adenocarcinoma was applicable. Altogether five studies focused on pulmonary adenocarcinoma, and the synthesized HR of OS was 2.220 (95% CI 1.607–3.066, *P* < 0.001). Fixed-effect model was applied to perform the analysis since heterogeneity was not significant (*P* = 0.781, *I*^2^ = 0%).

#### IHC Antibodies/H-Score

IHC was performed in all studies, and antibodies for MET staining varied between studies. As to seven studies that applied rabbit monoclonal antibodies, the combined HR for OS was 1.107 (95% CI 0.777–1.579, *P* = 0.573, *I*^2^ = 78.9%). Among which five studies adopted SP44 (Ventana Medical Systems, AZ, USA) antibody, and the pooled HR for survival was 1.031 (95% CI 0.668–1.590, *P* = 0.001, *I*^2^ = 78.1%). In addition, four studies via SP44 examined MET expression by same cut-off value with reference to methods by Spigel et al. (49), and the pooled HR was 1.031 (95% CI 0.668–1.590, *p* = 0.892). For the survival analysis of five studies that applied rabbit polyclonal antibodies, the pooled HR was 2.107 (95% CI 1.573–2.823, *P* < 0.001). Heterogeneity was not statistically significant (*p* = 0.521, *I*^2^ = 0%) thus fixed-effect model was preferred.

#### Primary Data

Ten studies addressed the prognostic role of MET over-expression among NSCLC by multivariate analysis. The pooled HR on OS was 2.004 (95% CI 1.229–3.268, *P* = 0.005). The remaining five studies were performed by univariate analysis, of which the pooled HR was 1.051 (95% CI 0.745–1.484, *p* = 0.776). Heterogeneity was significant among either results (*I*^2^ = 88.4 and 69.7, respectively). Therefore, random-effect model was adopted for both analyses.

**Figure 3:**
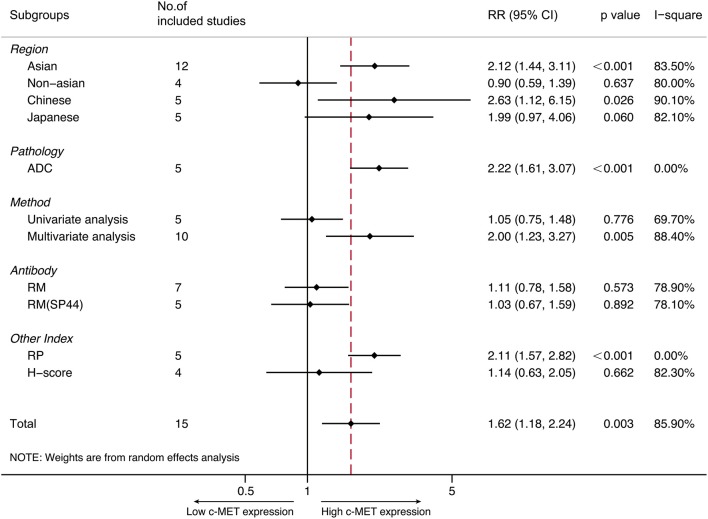
Forest plots representing the pooled results of subgroup analyses.

All summarized data was presented on Table 2 and shown in Figure 3.

**Table 2:** Meta-analyses of MET protein over-expression and survival of surgically resected NSCLC.

	***N* of studies**	**Model**	**HR (95% CI)**	**Log-rank p**	**Heterogeneity (*p*, *I*^**2**^)**	**Conclusion**
C-MET OS	15	Random	1.623 (1.176–2.240)	0.003	<0.001, 85.9%	Positive
P-MET OS	3	Random	1.710 (0.823–3.533)	0.15	0.006, 80.2%	Negative
Asian OS	12	Random	2.115 (1.440–3.108)	<0.001	<0.001, 83.5%	Positive
Non-Asian OS	4	Random	0.901 (0.586–1.387)	0.637	0.002, 80.0%	Negative
Japanese OS	5	Random	1.985 (0.970–4.058)	0.06	<0.001, 82.1%	Negative
Chinese OS	5	Random	2.627 (1.123–6.146)	0.026	<0.001, 90.1%	Positive
ADCC OS	5	Fixed	2.220 (1.607–3.066)	<0.001	0.781, 0%	Positive
RM OS	7	Random	1.107 (0.777–1.579)	0.573	<0.001, 78.9%	Negative
RM (SP44) OS	5	Random	1.031 (0.668–1.590)	0.892	0.001, 78.1%	Negative
H-score	4	Random	1.014 (0.822–1.251)	0.893	0.001, 0.893	Negative
RP OS	5	Fixed	2.107 (1.573–2.823)	<0.001	0.521, 0%	Positive
MVA OS	10	Random	2.004 (1.229–3.268)	0.005	<0.001, 88.4%	Positive
UVA OS	5	Random	1.051 (0.745–1.484)	0.776	0.010, 69.7%	Negative

*N, Number; HR, Hazard Ratio; CI, Confidence Interval; OS, Overall Survival; ADCC, Adenocarcinoma; RM, Rabbit Monoclonal; RP, Rabbit Polyclonal; MM, Mouse Monoclonal*.

#### Sensitivity Analysis and Publication Bias

**Figure 4:**
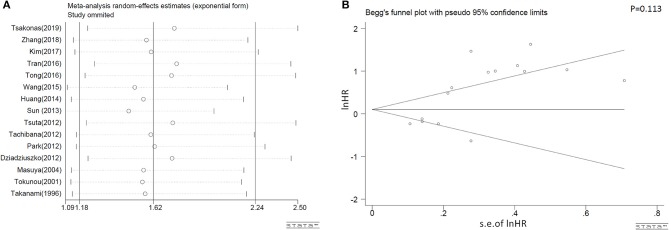
Sensitivity analyses results on omission of each individual study **(A)** and the Begg's publications plots **(B)** of eligible studies that assessed c-MET positivity and NSCLC survival on OS.

As shown in Figure 4A, the combined results representing the pooled HRs didn't prominently change when each study was sequentially removed, indicating the above synthesized results credible and robust. In addition, publication bias of our systematic review was neither found to exist, in accordance with Begg's plots in Figure 4B.

## Discussion

Our current study aimed to examine the prognostic role of c-MET/p-MET positivity among NSCLC patients that underwent surgical resection. With incorporated data, a meta-analysis was performed. As a result, although p-MET was not found to be associated with NSCLC survival, c-MET appears to be a prognostic factor that led to shorter OS. In view of Asian population, subgroup results indicated that c-MET was an inferior prognostic marker, and such is the same among Chinese people. Conversely, c-MET wasn't related with outcomes regarding Japanese participants. From a fixed-model, c-MET overexpression was significantly involved with inferior OS of patients with resected pulmonary adenocarcinoma. C-MET was in correlation with poor survival when rabbit polyclonal agents was applied for IHC, whereas neither rabbit monoclonal antibodies nor H-score were indicative when discussing its impact on survival of NSCLC whose c-MET was positive. Pooled result from univariate data suggested c-MET was not a marker of prognosis. On the contrary, synthesized data via multivariate analyses reflected a decisive conclusion which c-MET was an unfavorable prognostic marker of NSCLC.

From several aspects the adverse role of c-MET expression could be explained. Previous studies have noted that over-expression of c-MET was positively associated with vascular and lymphatic invasion, which led to higher risk of cancer relapse as well as more advanced stage among NSCLC patients (43, 50, 51). From therapy's experience, c-MET positivity was closely related with radio-resistance and chemo-resistance, hence correlated with unfavorable outcomes (52, 53). In terms of pathogenesis, HGF could facilitate tumor metastasis through MET/HGF pathways by inducing epithelial-mesenchymal transition (EMT) process of NSCLC (54, 55). And metastasis is considered as the major cause of lung cancer related death (56). In addition, c-MET over-expression was related with the prognosis of patients that harbored varied EGFR status as MET and EGFR shared signal molecules in downstream pathways (23, 46). Thus, MET over-expression could affect efficacy of patients that received EGFR-TKIs as a result (57). Interestingly, p-MET expression which represents activation level of MET didn't have an impact on survival of NSCLC in our study. As clinical research regarding p-MET is lacking (58), the prognostic role of p-MET remains to be further explored.

It remains controversial to determine how MET over-expressed. Alterations on transcription level of *MET* gene, which includes *MET* amplification and *MET exon 14* skipping mutation could be the potential mechanisms (21). Additionally, high gene copy number (GCN) of *MET* was also found be an adverse survival indicator in several studies (59–61). Nevertheless, MET positivity was notably higher, with a prevalence ranged up-till 70% among NSCLC, compared with *MET* mutations (around 4%) (58). With reference to previous studies, MET overexpression is positively correlated with NSCLCs that harbored *MET exon 14* skipping mutation and amplification both (20, 62). Indicating MET positivity could be adopted to screen NSCLC patients for further genetic profiling, as *MET* alterations has been recognized as a biomarker to receive Crizotinib treatment, and a potential trigger to cause first generation EGFR-TKIs resistance (19, 20, 63). In addition, MET over-expression was reported to be a favorable marker among NSCLC patients that received anti-MET therapy as an alternative. C-MET-positive patients had improved survival when given either anti-MET monoclonal antibodies (Onartuzumab) or MET inhibitors in combination with EGFR-TKIs, with reference to ongoing clinical trials (49, 64). Hence, understanding the nature of MET expression as well as establishing a standardized criteria regarding its evaluation, should be highlighted.

Previously two meta-analysis was published that assessed the impact of MET expression on survival among NSCLC (60, 65). Guo et al. integrated 13 studies and some of the results such as Asian/Non-Asian sub-group analyses were in concordance with ours. Yet a major concern of this systematic review was not making a distinction between c-MET and p-MET, as relevant literatures were combined as a whole. The other study by Pyo et al. also indicated that c-MET was an adverse prognostic factor, which is in agreement with ours, but merely 11 literatures were capable of data pooling. In addition, both systematic reviews adopted survival data via estimation from publications whose HR and CI were not directly provided. To avoid potential risk of bias, those literatures were excluded from our study. As numerous retrospective studies emerged in recent years, our systematic review with 17 publications incorporating 5,572 NSCLC patients has the largest data as well as information summarized in scale. To date it is the first systematic review that highlighted the impact of p-MET on NSCLC survival, as well as the first systematic review that analyzed the correlation between c-MET expression and NSCLC prognosis in many aspects such as IHC cut-off value and antibody selection.

Due to practical constraints, our meta-analysis has several limitations. Firstly, our several results had significant heterogeneity. Efforts such as sensitive analysis and subgroup analyses were performed on the basis of several aspects but a distinct source was still lacking. Hence we speculate that the existing heterogeneity could be attributed to the inconsistency of baseline characteristics from included literatures such as tumor stage, smoking status, post-operative therapies and IHC methodology involving varied cut-off values and antibody adoption. Tumor stage was highlighted in the protocol of the present study for its relationship with MET positivity, yet we failed to analyze the prognostic role of MET in each individual stage due to lack of original data. Moreover, explanations to the positive results derived from Asian population remains obscure. With respect to IHC, although several recent publications performed their IHC analysis with reference to an anti-MET clinical trial (49), a standardized criteria for IHC to determine MET positivity is lacking. Besides, we are unable to interpret the loss of survival when rabbit polyclonal antibodies were applied for MET IHC. Secondly, the amount of eligible literature in our study is relatively small, especially in the analysis of p-MET. Hence the current study could be re-conducted when more evidence have emerged. In addition to above, all data search in our study were carried out in English databases, hence some eligible publications written in other languages could have been neglected. Despite of limitations above, with discreetly pooled statistics and detailed protocols, bias was restrained to the minimum, and the results of the current study is guaranteed reliable.

## Conclusions

In conclusion, c-MET over-expression among resected NSCLC patients is a prognostic factor that indicated adverse survival on OS. Yet p-met didn't appear to have an impact on the prognosis of patients with NSCLC. The existing IHC criteria to define MET positivity is inconsistent, which might be a factor to cause heterogeneity. More studies should be conducted to examine the topic, especially studies that focuses on p-MET expression among NSCLC patients. The prognostic role of c-MET/p-MET both among NSCLC could be re-evaluated when added evidence have emerged by then.

## Data Availability Statement

The datasets generated for this study are available on request to the corresponding author.

## Author Contributions

GM and QZ conceived the study. GM and XL designed the study. GM and YD searched the literature and collected the data and performed the analyses. GM, YD, and WC drafted the manuscript. ZL and GM prepared Figures 1–4. CA and YD prepared Tables 1, 2. All authors reviewed and revised the manuscript.

### Conflict of Interest

The authors declare that the research was conducted in the absence of any commercial or financial relationships that could be construed as a potential conflict of interest.
